# REDD1 Is Essential for Optimal T Cell Proliferation and Survival

**DOI:** 10.1371/journal.pone.0136323

**Published:** 2015-08-24

**Authors:** Emma L. Reuschel, JiangFang Wang, Debra K. Shivers, Karuppiah Muthumani, David B. Weiner, Zhengyu Ma, Terri H. Finkel

**Affiliations:** 1 Division of Rheumatology, The Children's Hospital of Philadelphia, Philadelphia, Pennsylvania, United States of America; 2 Department of Pediatrics, University of Pennsylvania School of Medicine, Philadelphia, Pennsylvania, United States of America; 3 Department of Pathology and Laboratory Medicine, University of Pennsylvania School of Medicine, Philadelphia, Pennsylvania, United States of America; Jackson Laboratory, UNITED STATES

## Abstract

REDD1 is a highly conserved stress response protein that is upregulated following many types of cellular stress, including hypoxia, DNA damage, energy stress, ER stress, and nutrient deprivation. Recently, REDD1 was shown to be involved in dexamethasone induced autophagy in murine thymocytes. However, we know little of REDD1’s function in mature T cells. Here we show for the first time that REDD1 is upregulated following T cell stimulation with PHA or CD3/CD28 beads. REDD1 knockout T cells exhibit a defect in proliferation and cell survival, although markers of activation appear normal. These findings demonstrate a previously unappreciated role for REDD1 in T cell function.

## Introduction

Regulated in development and DNA damage response 1 (REDD1), also referred to as Dig2, RTP801, and DDIT4, is a highly conserved stress response gene that is upregulated following many types of cellular stress. It was first identified as being upregulated by hypoxia [1] and DNA damage [2] in mouse embryonic stem cells and fibroblasts. Soon after, it was found to be upregulated in murine thymocytes following treatment with dexamethasone, a glucocorticoid [3]. It has since been discovered to be upregulated in many cell types under other forms of stress, including energy stress [4], ER stress [5,6], nutrient deprivation [7], gamma radiation [8], serum deprivation [9], and stress related catecholamine treatment [10]. REDD1 was identified by our group in a screen for genes that were upregulated in response to the stress of HIV infection [11].

Significantly, REDD1 has been shown to function as an inhibitor of mammalian target of rapamycin (mTOR), specifically mTOR complex 1 (mTORC1) in several cell types [4,5,7,9,12–17]. The mTOR signaling pathway is a key regulator of cell growth, proliferation, and survival by integrating various signals about the stress, or lack thereof, a cell is currently experiencing [18–21]. It has emerged as a central regulator of immune responses [22], including regulating T cell activation vs. anergy [23]. REDD1 is thought to inhibit the interaction of 14-3-3 with tuberous sclerosis complex 2 (TSC2), thus activating the TSC1/2 complex and allowing it to inhibit mTORC1[16]. Molitoris *et al*. [24] have shown that REDD1 also functions as an inhibitor of the mTOR pathway in thymocytes, leading to an induction of autophagy as a survival mechanism following dexamethasone treatment. REDD1 has also been shown to regulate reactive oxygen species (ROS) [1,2,25], though the mechanism of this regulation remains unidentified.

Though REDD1 has been shown to be upregulated in thymocytes in response to treatment with dexamethasone, a stress hormone, very little is known about REDD1 in mature T cells. Upon activation via the T cell receptor and costimulatory molecules, mature naive T cells undergo massive metabolic changes in order to proliferate and perform effector functions such as cytokine secretion [26]. These changes result in significant stress on the activated T cell. mTOR is key to integrating signaling involved in all of these processes and, therefore, in determining the characteristics of the resulting immune response. Because of REDD1’s previously shown role in regulating mTOR activity, we investigated the role of REDD1 in mature T cell activation. We demonstrate for the first time that REDD1 is upregulated in T cells stimulated with phytohemagglutinin (PHA) or CD3/CD28 beads. T cells from REDD1 knockout mice show a decrease in T cell proliferation and survival, although they are able to normally upregulate the activation markers CD69 and CD25. This suggests a role for REDD1 in helping mature T cells to manage the stress of activation, perhaps through the induction of autophagy as a survival mechanism as has been shown in dexamethasone treated thymocytes [24].

## Materials and Methods

### Ethics Statement

All mouse work was carried out in strict accordance with the recommendations in the Guide for the Care and Use of Laboratory Animals of the National Institutes of Health. The protocol was approved by the Children’s Hospital of Philadelphia Institutional Animal Care and Use Committee (protocol number: 449). De-identified human primary T cells were obtained from the University of Pennsylvania's Human Immunology Core. Secondary use of de-identified human specimens by individual Core users is not considered human subjects research by the National Institutes of Health or the University of Pennsylvania's Institutional Review Board.

### REDD1 Knockout Mice

REDD1 knockout mice [27] were backcrossed onto the C57BL6 background (Jackson Labs) for 6 generations. Age-matched wildtype and knockout mice between 2 and 4 months old were used. Spleens and lymph nodes (inguinal, axillary, brachial, and cervical) were removed and mashed through a 40 μm nylon cell strainer (Falcon). The cells were washed with media and then counted on a Countess automated cell counter (Invitrogen).

### Cell culture

Primary human CD4 T cells were obtained from the University of Pennsylvania’s Human Immunology Core. Human CD4 T cells were maintained in RPMI (Gibco) with 10% FBS (Benchmark), 100 U/ml Penicillin-Streptomycin (Gibco), and 2 mM L-glutamine (Gibco). Mouse cells were maintained in Click's media (Irvine Scientific) with 10% FBS, 100 U/ml Penicillin-Streptomycin, 2 mM L-glutamine, 1 mM sodium pyruvate (Cellgro), 1x MEM-NEAA (Gibco) and 50 μM 2-mercaptoethanol (BioRad).

### Activation and cell survival assays

Cells were plated at 1 million cells/ml in media, and then stimulated with the indicated concentrations of Human T Activator CD3/CD28 beads (Dynal) or PHA (Remel) and IL-2 (NIH AIDS Research and Reference Reagent Program). For proliferation assays, cells were resuspended at 2 million cells/ml in phosphate buffered saline (PBS) (Gibco) with 0.1% FBS. A 20 μM solution of 5(6)-Carboxyfluorescein diacetate N-succinimidyl ester (CFSE) (Sigma) in PBS with 0.1% FBS was mixed 1:1 with the cell suspension, and incubated for 15 minutes at room temperature in the dark. The labeling was stopped by adding 5 volumes of cold media and incubating on ice for 5 minutes. Cells were then stimulated with PHA and IL-2 as indicated. For cytokine production assays, T cells were first purified from total lymph node cells using the EasySep Negative Selection Mouse T Cell Isolation Kit (StemCell Tech) as directed, then stimulated as in the activation and cell survival assays.

### Flow cytometry

Flow cytometry data was collected on a FACS Fortessa (BD) and analyzed using FlowJo (Treestar). The following antibodies and reagents were used: LIVE/DEAD Fixable Dead Cell Stain (Invitrogen), CD3-BV421 (BioLegend), CD4-BV711 (BioLegend), CD8-BV605 (BioLegend), CD19-FITC (BD Pharmingen), CD69-PE-Cy7 (BD Pharmingen), CD25-PE-CF594, (BD Horizon), Annexin V-APC (BD Pharmingen), and propidium iodide (PI) (BD Pharmingen). For proliferation and activation assays, cells were washed once with PBS, and then incubated with LIVE/DEAD Fixable Dead Cell Stain in PBS for 30 minutes at room temperature in the dark. After washing with PBS with 1% FBS, cells were incubated with surface marker antibodies in PBS with 1% FBS for 20 minutes at room temperature in the dark. The cells were then washed and resuspended in PBS with 1% FBS, and the samples were run on the flow cytometer. For Annexin V and PI staining, cells were washed once with binding buffer (eBioscience), then incubated with Annexin V and cell surface markers in binding buffer for 15 minutes at room temperature in the dark. After washing with binding buffer, cells were resuspended in binding buffer with PI, and the samples were analyzed by flow cytometry.

### Quantitative reverse transcription-polymerase chain reaction (qRT-PCR)

Total RNA was extracted using the RNA/DNA/Protein Purification Kit (Norgen). cDNA was reverse transcribed from 0.1 μg of RNA using the High-Capacity cDNA Reverse Transcription Kit (Applied Biosystems). qPCR was performed in 20 μl reactions containing 1 μl of cDNA, 1 μl of the appropriate primer/probe set, 8 μl of TaqMan Universal Mastermix, No AmpErase UNG (Applied Biosystems), and 10 μl of PCR-grade water (Fisher). All samples were run in triplicate. The following primer/probe sets were purchased from Applied Biosystems: human REDD1 (Hs01111686_g1), mouse REDD1 (Mm00512504_g1), and RN18S1 (Hs03928990_g1). qPCR reactions were run on an SDS 7500 Real-time PCR system (Applied Biosystems) with the following protocol: 1 cycle of 95°C for 10 minutes; 40 cycles of 95°C for 15 seconds followed by 60°C for 1 minute. REDD1 expression levels were normalized to the endogenous control, RN18S1. RN18S1 was identified as stably expressed in our system from among the other commonly used housekeeping genes RPL13A, TBP, B2M, and GAPDH using GeNorm, a Visual Basic Application for Excel developed by Vandesompele *et al* [28].

### Immunoblotting

Protein was extracted using the RNA/DNA/Protetin Purification Kit (Norgen). Protein concentration was measured using Coomassie Plus (Pierce). Equal amounts of total protein were mixed with sample buffer (Invitrogen) and reducing agent (Invitrogen). Samples were heated at 70°C for 10 minutes and loaded onto a 4–12% Bis-Tris NuPAGE gel (Invitrogen). Gels were run on a NuPAGE electophoresis system (Invitrogen) at 200 V for 50 minutes in MOPS running buffer. Samples were then transferred onto an Immobilon-FL PVDF membrane (Millipore) at 30 V for 1 hour and blocked with Odyssey blocking buffer (LiCore). The following antibodies were used: rabbit-anti-REDD1 (Proteintech), mouse-anti-actin (Sigma), goat-anti-rabbit-IRDye 800CW (LiCor) and goat-anti-mouse-IRDye 680RD (LiCor). The membranes were imaged on the Odyssey CLx (LiCor) and analyzed using the Image Studio software (LiCor).

### Statistics

Prism software was used to perform two-way analysis of variance for data with multiple timepoints and unpaired t-tests for data from a single timepoint. Error bars show the standard error of the mean. P values less than 0.05 were considered significant.

## Results

### REDD1 is upregulated in human and mouse T cells upon stimulation with PHA or CD3/CD28 beads

**Fig 1:**
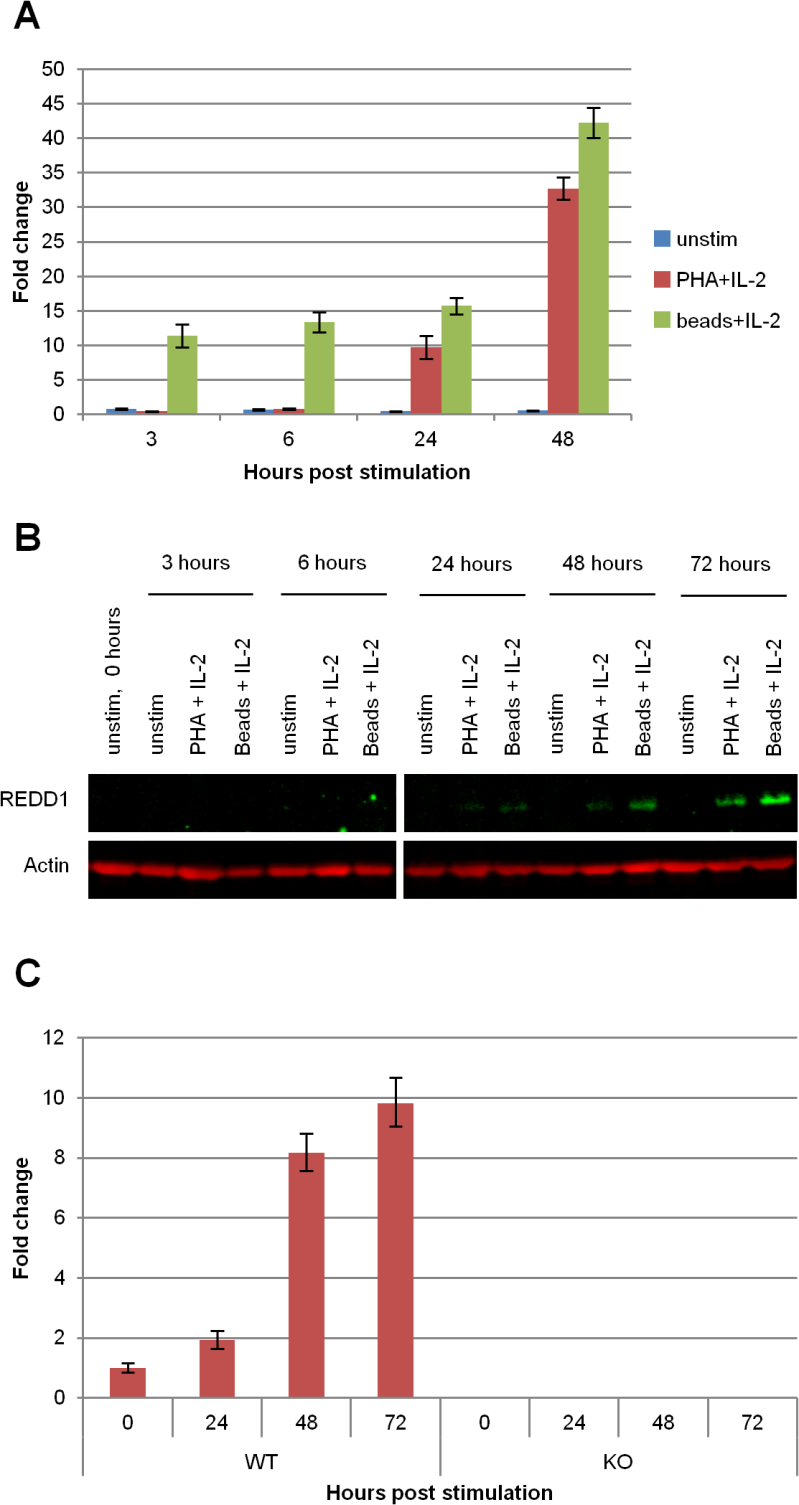
REDD1 mRNA and protein is expressed in lymphoid tissues and is upregulated during T cell activation. Primary human CD4 T cells were stimulated with 1.5 μg/ml PHA + 100 U/ml IL-2 or 3 CD3/CD28 beads/cell + 100 U/ml IL-2. REDD1 mRNA **(A)** and protein **(B)** expression was determined using qRT-PCR and immunoblot, respectively. **(C)** Mouse splenocytes were stimulated with 1.5 μg/ml PHA + 20 U/ml IL-2 and REDD1 mRNA expression was determined by qRT-PCR. All qRT-PCR data is presented as fold change compared to the unstimulated cells. qRT-PCR and immunoblot data are representative of 3 individual experiments.

To study the role of REDD1 in normal T cell function, we first determined the level of REDD1 expression in primary human CD4 T cells in response to activation signals. REDD1 mRNA was significantly upregulated by PHA and beads coated with anti-CD3 and anti-CD28 antibodies (Fig 1A). CD3/CD28 beads induced a 10-fold increase in REDD1 mRNA as early as 3 hours after stimulation, and reached a 40-fold increase at 72 hours. In comparison, PHA induced REDD1 mRNA upregulation at a later time and at a lower level. REDD1 protein expression increased accordingly (Fig 1B). An increase in REDD1 mRNA was also detected in mouse splenocytes stimulated with PHA (Fig 1C). Similar to human T cells, the increase was most pronounced at 48 and 72 hours.

### REDD1 is required for optimal T cell proliferation

**Fig 2:**
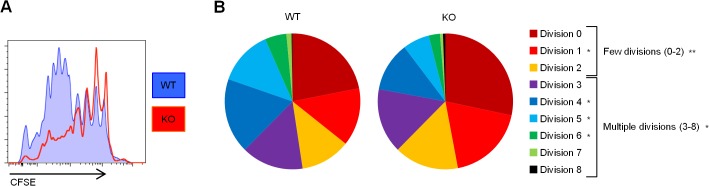
REDD1 is required for optimal T cell proliferation. Wildtype (WT) and knockout (KO) mouse lymph node cells were labeled with CFSE and stimulated with 1.5 μg/ml PHA for 72 hours. **(A)** Representative flow plots of CFSE staining gated on CD4 T cells. **(B)** Average percentages of CD4 T cells that have undergone the indicated number of divisions in 72 hours. Statistically significant differences between the WT and KO populations for each division are indicated next to the legend. Statistically significant differences between 'few divisions' (divisions 0–2) and 'multiple divisions' (divisions 3–8) are also indicated. N = 8 WT; N = 8 KO. *p = 0.01 to 0.05; **p = 0.001 to 0.01; ***p < 0.001.

The pronounced upregulation of REDD1 mRNA and protein upon stimulation led us to explore its potential role in T cell activation. To determine the role of REDD1 in T cell proliferation, we took advantage of REDD1 knockout mice [27]. The lack of REDD1 expression in knockout splenocytes was confirmed using qRT-PCR (Fig 1C). In a CFSE-based proliferation assay, CD4 T cells from the REDD1 knockout mice showed impaired proliferation in comparison to wildtype mice 72 hours after PHA stimulation (Fig 2A). A lower percentage of knockout CD4 T cells were able to divide multiple times compared to the wildtype CD4 T cells. Further analysis of the number of cells in each division using FlowJo’s proliferation analysis platform revealed that REDD1 is correlated with the capacity of T cells to undergo multiple rounds of division (Fig 2B). The REDD1 knockout T cells were more likely to go through 2 or fewer divisions, while the majority of wildtype T cells went through 3 or more divisions within the same period of time.

### REDD1 does not affect upregulation of T cell activation markers

**Fig 3:**
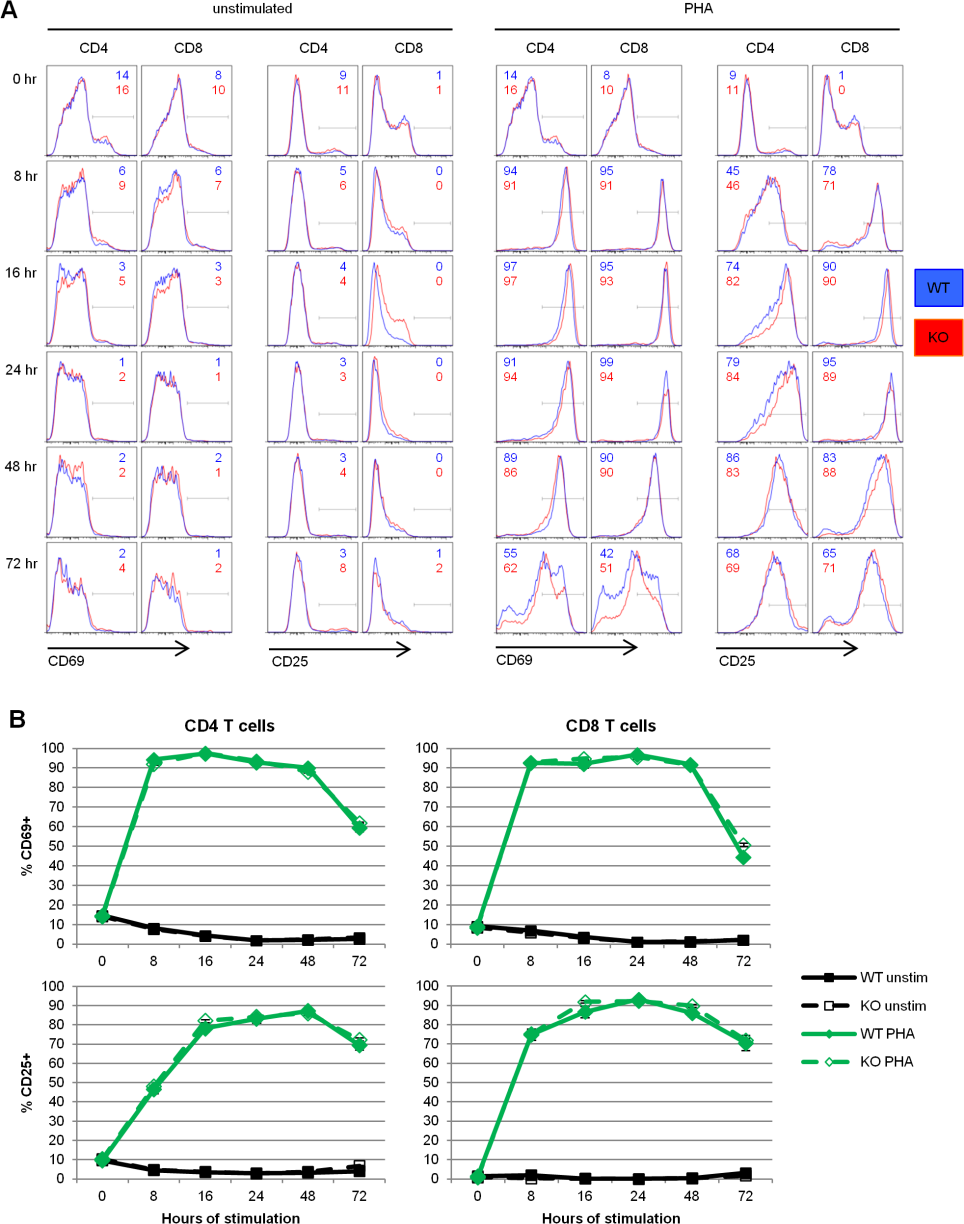
REDD1 does not affect upregulation of activation markers CD69 or CD25. Wildtype (WT) and knockout (KO) mouse lymph node cells were stimulated with 1.5 μg/ml PHA and CD69 and CD25 expression was measured by flow cytometry. **(A)** Representative flow plots of CD69 and CD25 staining gated on CD4 or CD8 T cells. The percentage of WT and KO cells falling within the gate are indicated in the corner of each panel. **(B)** Average percentages of CD4 or CD8 T cells expressing CD69 and CD25 after stimulation. N = 4 WT; N = 4 KO.

To determine the role of REDD1 in other aspects of T cell activation, we compared the upregulation of activation markers CD69 and CD25 by wildtype and REDD1 knockout T cells in response to PHA stimulation. CD4 and CD8 T cells from REDD1 knockout mice and wildtype controls displayed closely matched patterns of CD69 and CD25 expression during the course of a 72 hour stimulation (Fig 3A). Statistical analysis of the percentage of T cells with upregulated CD69 or CD25 of four replicate experiments did not reveal any significant differences between REDD1 knockout and wildtype T cells (Fig 3B). The apparent normal T cell activation marker expression in REDD1 knockout T cells suggest that REDD1 does not play a role in early T cell activation.

### REDD1 is required for optimal cell survival, independent of stimulation

**Fig 4:**
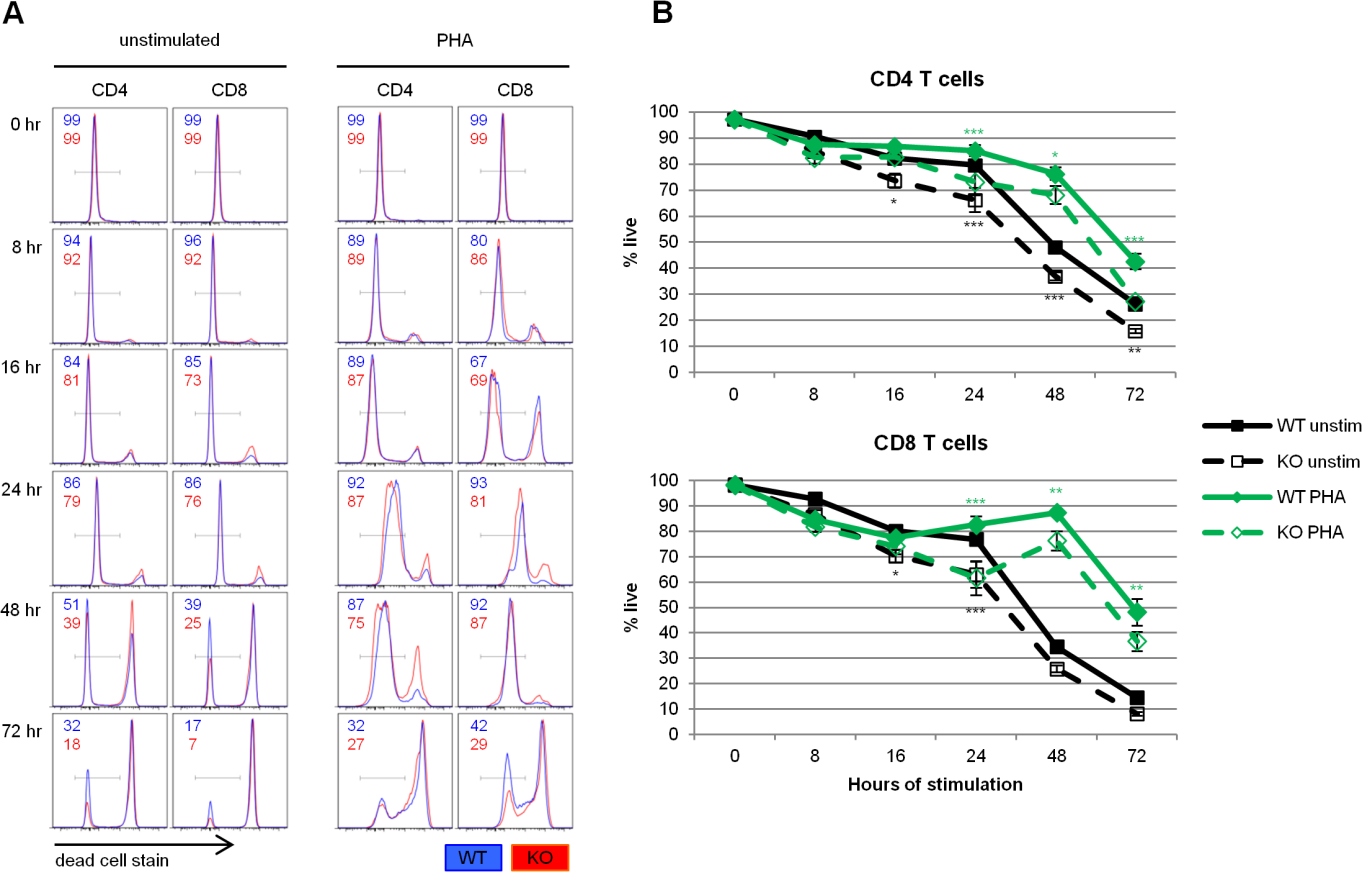
REDD1 is required for optimal cell survival independent of stimulation. Wildtype (WT) and knockout (KO) mouse lymph node cells were stimulated with 1.5 μg/ml PHA and cell survival was measured by flow cytometry with dead cell staining. **(A)** Representative flow plots of dead cell staining, gated on CD4 or CD8 T cells. The percentage of WT and KO cells falling within the gate are indicated in the corner of each panel. **(B)** Average percentages of live CD4 or CD8 T cells. N = 8 WT; N = 8 KO. *p = 0.01 to 0.05; **p = 0.001 to 0.01; ***p < 0.001.

Our lab first became interested in the role of REDD1 in T cells with our report of REDD1 upregulation in HIV-infected human T cells that are resistant to apoptosis [11]. The role of REDD1 in regulating apoptosis was further described in other studies [1,29]. To investigate the role of REDD1 in T cell survival, we first evaluated the number of PHA stimulated T cells that stained positive for a dead cell stain, an indicator of loss of plasma membrane integrity. REDD1 knockout CD4 and CD8 T cells were more likely to die than their wildtype counterparts, whether they had been stimulated with PHA, or not (Fig 4A and 4B). As early as 16 hours after *ex vivo* culture, the unstimulated knockout CD4 and CD8 T cells had a statistically significant reduction in the percentage of live cells compared to wildtype cells. As early as 24 hours after stimulation, the knockout CD4 and CD8 T cells stimulated with PHA also exhibited a statistically significant reduction in the percentage of live cells.

**Fig 5:**
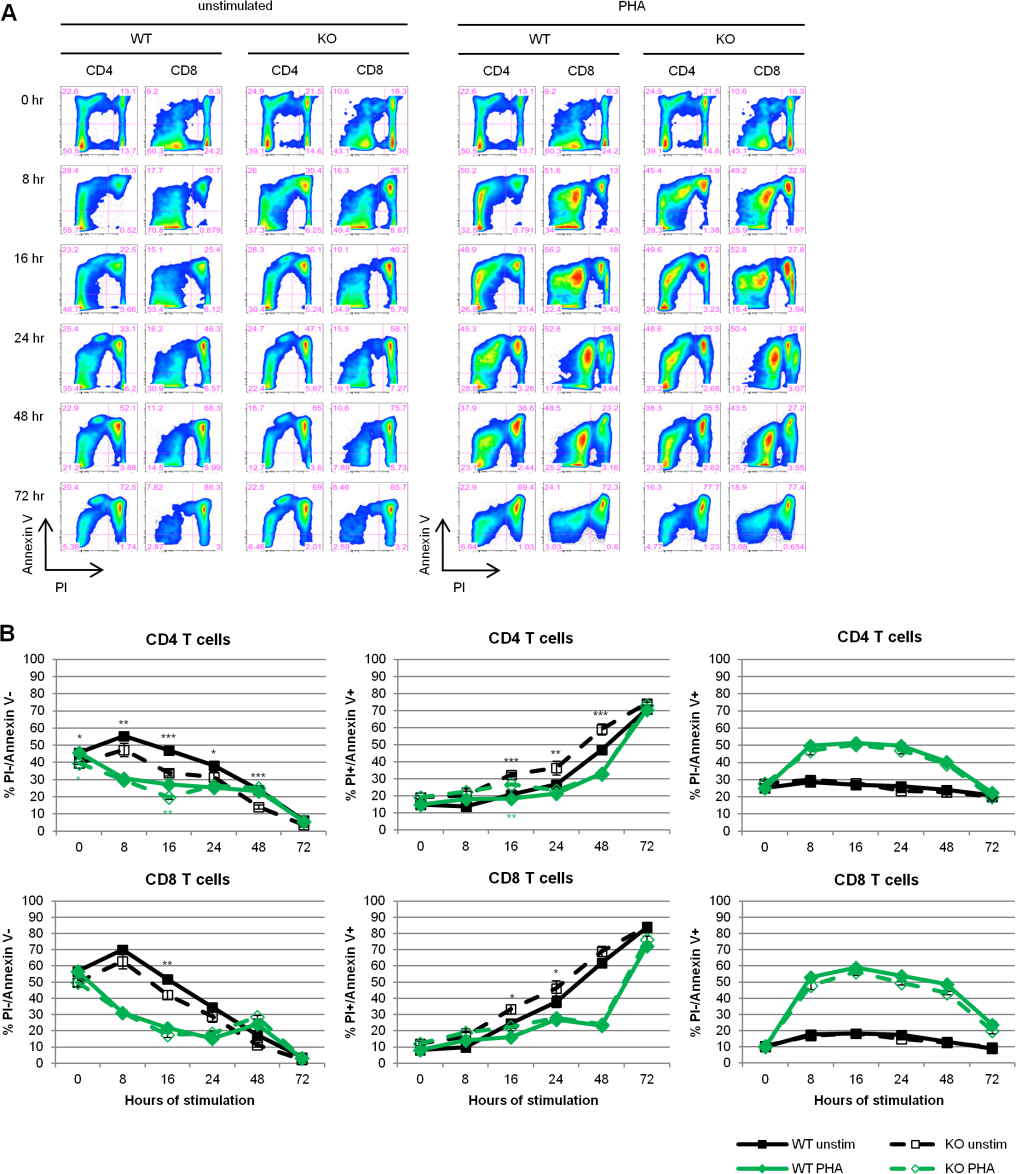
REDD1 does not affect PHA induced apoptosis. Wildtype (WT) and knockout (KO) mouse lymph node cells were stimulated with 1.5 μg/ml PHA and cell survival was measured by flow cytometry with PI/Annexin V staining. **(A)** Representative flow plots of Annexin V/PI staining gated on CD4 or CD8 T cells. **(B)** Average percentages of live (PI-/Annexin V-), dead (PI+AnnexinV+), and apoptotic (Annexin V+) CD4 or CD8 T cells. N = 4 WT; N = 4 KO. *p = 0.01 to 0.05; **p = 0.001 to 0.01; ***p < 0.001.

To confirm these results, we repeated the same set of experiments with PI/Annexin V staining as another measure of cell viability. Live cells are PI-/Annexin V-. As cells undergo apoptosis, they expose phosphatidylserine (PS) on the outside of their plasma membrane, allowing Annexin V to bind, and become Annexin V+. At later timepoints, apoptotic cells lose membrane integrity and become PI+/Annexin V+. Necrotic cells, however, directly become PI+/Annexin V+ [30]. Because we cannot discriminate between necrotic and late apoptotic dead cells, we refer to PI+/Annexin V+ cells simply as dead. By measuring early apoptosis over a time course, we expected to catch any changes in this population over time. With this measurement, the effect of REDD1 on cell survival is most pronounced in the unstimulated CD4 T cells (Fig 5A and 5B). There were significantly fewer PI-Annexin V-, or live, unstimulated REDD1 knockout CD4 T cells at all timepoints except 72 hours and a complementary significant increase in PI+Annexin V+, or dead, unstimulated knockout CD4 T cells as early as 16 hours after plating. REDD1 knockout CD8 T cells also showed a significant decrease in live unstimulated cells and an increase in dead unstimulated cells as early as 16 hours after plating. With PHA stimulation, only the CD4 T cells showed a significant decrease in live cells and concomitant increase in dead cells at any time point. Interestingly, the absence of REDD1 did not appear to have an effect on apoptosis of PHA stimulated or unstimulated CD4 or CD8 T cells (Fig 5B). In total, these experiments demonstrate a protective role for REDD1 in T cell survival independent of stimulation, though REDD1 does not appear to regulate PHA induced apoptosis.

## Discussion

REDD1 is highly conserved, and is known to be upregulated following many stresses, including DNA damage, hypoxia, and dexamethasone treatment [1–3]. Here, we show that REDD1 is also upregulated following T cell stimulation by CD3/CD28 beads or PHA in primary human CD4 T cells (Fig 1A and 1B) and primary mouse splenocytes (Fig 1C). To our knowledge, this is the first demonstration of REDD1 elevation following T cell activation.

In order to study the role of REDD1 in mature T cells, we took advantage of a previously developed REDD1 knockout mouse [27] as a source of REDD1 knockout T cells. While these REDD1 knockout mice have no obvious immunological phenotype to point to REDD1’s importance in T cells, laboratory mice are housed in animal facilities with the specific purpose of preventing exposure to pathogens, which may account for the apparent lack of phenotype.

REDD1 is a stress response gene that has been shown to function as an inhibitor of mTOR in several cell types, including thymocytes stimulated with the stress hormone dexamethasone [24]. It has also been shown to regulate reactive oxygen species (ROS) in several cell types [1,2,25]. Both mTOR signaling [23] and ROS [31] have been shown to be important in T cell activation and proliferation. These connections, along with REDD1’s upregulation following T cell stimulation, led us to investigate what role REDD1 might be playing in the activation and proliferation of T cells. While REDD1 knockout T cells were capable of proliferating, we observed a defect in the ability of REDD1 knockout CD4 T cells to undergo multiple rounds of division in response to PHA stimulation compared to their wildtype counterparts (Fig 2).

To determine if REDD1 knockout T cells were capable of being normally activated, we looked at the effect of PHA stimulation on the upregulation of activation markers on wildtype and knockout T cells. Neither CD69 nor CD25 expression after PHA stimulation was affected by the lack of REDD1 over the same time period as the defect in proliferation was observed (Fig 3). The normal upregulation of CD69 and CD25 suggests that the absence of REDD1 does not affect the ability of CD4 or CD8 T cells to activate in response to PHA stimulation.

We next looked at REDD1’s effect on T cell survival. While REDD1 expression has been shown to have either a pro- or anti-survival effect in different cell types [1,27], in developing lymphocytes, it has been shown to be pro-survival. REDD1 knockout primary mouse thymocytes are more sensitive to dexamethasone induced cell death [24], and REDD1 overexpression in a mouse T cell lymphoma cell line is protective [3]. Our lab has previously identified REDD1 as being upregulated in non-apoptotic HIV infected CEM-SS cells compared to apoptotic infected cells [11], also pointing to REDD1’s pro-survival properties in T cells. Here, we show that REDD1 knockout CD4 and CD8 T cells exhibit a survival defect, both when they are stimulated with PHA, and when left unstimulated (Fig 4A and 4B). These data are consistent with previous reports of REDD1's pro-survival effect in lymphocytes.

In order to better understand what type of cell death REDD1 is involved in regulating, we used PI/Annexin V staining to measure apoptosis of REDD1 knockout cells. With PI/Annexin V staining, early apoptotic cells are Annexin V+, while late apoptotic and necrotic cells are PI+/Annexin V+. Because this method is unable to discriminate between late apoptotic and necrotic cells, we refer to them simply as dead cells. In an effort to catch any effect of REDD1 on early apoptosis, we took measurements at several timepoints following stimulation. The PI/Annexin V staining shows a decrease in live unstimulated REDD1 knockout CD4 and CD8 T cells and a corresponding increase in dead cells (Fig 5B), supporting our previous data simply measuring total cell viability. With PHA stimulation, only CD4 T cells showed an effect of REDD1 on the number of dead cells (Fig 5B). Interestingly, REDD1 does not appear to have an effect on the apoptosis of unstimulated or PHA stimulated CD4 or CD8 T cells (Fig 5B). While REDD1 has been shown to exert a pro-survival effect in thymocytes by protecting them from apoptosis following DNA damage [32], it has also been shown to have no effect on apoptosis in dexamethasone treated thymocytes [33]. This highlights the potential differences in REDD1 function, not only in different cell types, but also under different stimulation conditions. REDD1’s apparent lack of effect on the apoptosis of PHA stimulated or unstimulated T cells cultured *ex vivo*, leads us to conclude that REDD1 protects mature T cells from another form of cell death.

It has recently become clear that a type of regulated necrosis linked to autophagy called necroptosis, is separate from, and cross-inhibitory to, apoptosis. Caspase 8 deficient T cells stimulated with anti-CD3 and CD28 antibodies have been shown to die by necroptosis [34]. Interestingly, necroptosis has been found to involve the generation of high levels of ROS [35], providing another potential link to REDD1. Necroptosis appears to be intimately tied to autophagy through the receptor-interacting serine/threonine-protein kinase 1 (RIPK1) [36]. Autophagy is known to be important for multiple aspects of T cell function, from the normal homeostatic turnover of damaged organelles, to the recycling of defective mitochondria during the intense metabolic changes following activation [37,38]. In fact, T cells unable to undergo autophagy due to genetic deletion of the Atg5 gene present a remarkably similar phenotype to REDD1 deficient T cells [39]. They are unable to proliferate efficiently, show normal CD69 and CD25 expression upon activation, and are more susceptible to cell death. This, along with REDD1’s reported involvement in autophagy in dexamethasone treated thymocytes [24], makes the future investigation of REDD1’s involvement in autophagy in activated T cells of great interest.

In summary, we show for the first time that REDD1 is upregulated during T cell activation. We also show that the absence of REDD1 causes a decrease in the proliferation of PHA stimulated T cells and a decrease in survival of stimulated and unstimulated T cells, but does not appear to significantly affect T cell activation. Further study is needed to elucidate the biochemical mechanisms of these effects of REDD1 on T cell homeostasis.
